# The 17^th^ SSNN International Summer School of Neurology 2022 – Part 2

**DOI:** 10.25122/jml-2022-1024

**Published:** 2022-09

**Authors:** Stefana-Andrada Dobran, Alexandra-Mihaela Gherman, Dafin Fior Muresanu

**Affiliations:** 1 RoNeuro Institute for Neurological Research and Diagnostic, Cluj-Napoca, Romania; 2 Sociology Department, Babes-Bolyai University, Cluj-Napoca, Romania; 3 Department of Neuroscience, Iuliu Hatieganu University of Medicine and Pharmacy, Cluj-Napoca, Romania

The first part of the SSNN Summer School of Neurology 2022, which covered the first day's events, is highlighted in a previous publication.

The second day of the congress was introduced by **Natan Bornstein (Israel)** and Dafin Muresanu (Romania). The beginning session of the day, Session 5, started with Natan Bornstein (Israel), the Director of the Brain Division and Chairman of the Israeli Stroke Society (ISS), and his two presentations: "Secondary Stroke Prevention – The DAPT Story – A Journey of 25 Years" and "Post-stroke cognitive decline – current concepts and treatment approaches". Prof. Bornstein firstly stated the importance of tertiary prevention of stroke with the syntagm "don't let stroke strike twice", as stroke represents a major cause of long-term disability, and went further into discussing current guidelines and the long-term story of Dual Antiplatelet Therapy (DAPT) research, beginning back in 1996. His second presentation discussed treatment options following ischemic stroke, highlighting the lessons from double-blind randomized trials in patients with acute mild ischemic stroke or high-risk Transient Ischemic Attack (TIA). Moreover, as post-stroke depression (PSD) represents the most frequent non-cognitive neuropsychiatric complication and is associated with higher mortality, poorer recovery and decreased quality of life, the investigation of its mechanisms remains a subject of utmost importance. Due to this, approaches should be multidimensional, involving a multidisciplinary team for the best results.

**Dafin Muresanu (Romania)**, President of the The European Federation of NeuroRehabilitation Societies (EFNR), presented microcirculation and stroke and discussed the 3 crucial pillars in neurorehabilitation and brain protection and recovery:

1. Developing a solid theory and strong hypothesis, validating the ideas with basic research;
2. Testing the effectiveness of the intervention;
3. Using evidence-based parameters.

He then pinpointed two critical aspects of rehabilitation: the limit of the disability and the individual biological reserve. In addition, the importance of creating promising paradigms and protocols for treatments and aligning the mechanisms of action with the design was showcased, and the two anticorrelated sequences of post-lesional brain regulation (NEUROPROTECTION and NEURORECOVERY) were also highlighted. Moreover, Prof. Muresanu discussed endogenous defence activity, damage mechanisms, and the levels of central nervous system (CNS) endogenous modulation. One crucial point touched upon was the importance of the proportion and interactions as more relevant factors than the separate effects of each function. Furthermore, he offered an in-depth view of the anticorrelated processes of 3 different levels, discussing the vertical correlation between functional and gene networks. The principal therapeutic approach in acute ischemic stroke was also outlined, which refers to:

- Arterial revascularization;
- The substantial predictive effect on clinical outcomes of the final recanalization;
- The factors on which brain tissue perfusion depends.

In his comprehensive presentation, he discussed the combination of approved methods, avenues for enhancing thrombolysis, and the role of Cerebrolysin in inhibiting endogenous inflammatory pathways. Prof. Muresanu closed the session with a lesson on performing neurorestorative treatments with extended therapeutic windows and recommended that specialists work together in integrative approaches, highlighting the role of the European Federation of NeuroRehabilitation Societies (EFNR).

The 6^th^ session, hosted by Dafin Muresanu and Adina Stan, began with the presentation of **Jesse Dawson (UK)**, Professor of Stroke Medicine at the Institute of Cardiovascular and Medical Sciences in Glasgow (UK) on "Stimulation (neuromodulation) for post-stroke recovery". Prof. Dawson highlighted that there are many mechanisms for upper limb impairment, therefore, many avenues for recovery. As a result, the interventions should be related to the cause of impairment. The approach in stroke rehabilitation refers to causing task-specific plasticity, as neuro-connectivity and activity are altered post-stroke. Also, with plasticity being task-specific, neuromodulation – the alteration of neuro-activity to target the delivery of a stimulus to specific neurological sites – encompasses both advantages and disadvantages, showcased in his presentation. Prof. Dawson discussed different types of stimulation and their respective roles and effects, as well as approached VNS (vagus nerve stimulation) after ischemic stroke for upper limb motor function, highlighted in his research.

**Andreas Winkler (Austria)**, Medical Director and Head of Department in Neurological Rehabilitation at Klinik Bad Pirawarth in Vienna (Austria), presented "How can we be more efficient in motor recovery after stroke?". Prof. Winkler discussed the possibility of improving endogenous plasticity and introducing a new window of opportunity to obtain a better neurological outcome for the patient. He approached the enhancement of post-stroke plasticity and the limitations of current undertakings, including selective serotonin reuptake inhibitors (SSRIs), and discussed three clinical trials to highlight his previous point. Moreover, he showcased the usefulness of Cerebrolysin in stroke patients as outlined by different medical societies and the shift from past approaches to this innovative agent, underlining the positive effects of Cerebrolysin on neuroplasticity. The role of Brain-derived neurotrophic factor (BDNF) as a biomarker in stroke and the enhancement of post-stroke plasticity through electrical stimulation were also tackled. One important point of his presentation was combining different approaches to boost plasticity.

**Adina Stan (Romania)**, Primary Neurologist and Lecturer of Neurology at the University of Medicine and Pharmacy Iuliu Hatieganu in Cluj-Napoca (Romania), presented a case study on stroke, showcasing the disease progression and process of examination, discussing the affections of the patient, and engaging the public to suggest the most probable diagnosis based on the available information. She also tackled the differential diagnosis, discussed the TOAST classification of stroke subtypes and underlined the role of stroke prevention. Following her presentation, **Hanna Dragos (Romania)**, from Cluj-Napoca Emergency County Hospital (Romania), offered an inspiring approach to a rare disease, discussing the symptoms, disease progression, medical history examinations, and, similarly, asked for public input on the most likely diagnosis. Finally, after covering multiple aspects, she touched upon the molecular genetic diagnostic pathway for stroke-like episodes and the management of the disease.

During the 7^th^ session, presided by Cristina Tiu (Romania) and Rodica Balasa (Romania), **Cristina Tiu (Romania)**, Head of the II Neurology Department and Head of the Neurology Discipline at the Neurology Clinic from the Bucharest University Emergency Hospital in Bucharest (Romania), introduced the public to her presentation "About immunosenescence and inflammaging in multiple sclerosis (MS). Therapeutic options in older patients". With elderly patients being a challenging population and real-life patients differing from those presented in clinical studies, complex and multimodal approaches to diagnostics and treatment are needed. Prof. Tiu discussed the prevalence of MS and various epidemiological characteristics, the immunosenescence of innate immune systems, the meningeal inflammation, and the influence of age on the pathophysiology of MS and imaging features for identifying comorbidities. Two scenarios were tackled: (1) diagnosis at older age *vs*. (2) patients with known MS who are aging naturally, with particularities detailed for each. Finally, she argued whether the discontinuation of immunomodulatory therapy is recommended in patients over 60 years of age from a therapeutic perspective, pinpointing the aspects of biological age, epigenetic factors, and lifestyle strategies.

**Mihaela Simu (Romania)**, Professor and Chairman of the Neurology Department II – the University of Medicine and Pharmacy Victor Babes in Timișoara (Romania), presented "Cognitive impairment in MS – from theory to practice". Historical notes, statistical facts, and the science of cognition and neuropsychology were discussed first. The presentation then focused on statistical data on cognitive deficits in MS and various neuropsychiatric aspects of the disease, with an outline of the neuropsychological patterns in MS cognitive impairment according to phenotypes and the similarities of cognitive profiles of different MS phenotypes. Further on, Prof. Simu offered a view into the types of cognitive deficits, assessment of MS, and the prevalence of cognitive deficits in MS depending on the subtype, mentioning the pediatric population. The classification of MS and the various tests and screening methods were also discussed, and the presentation ended with recommendations for cognitive impairment (CI) evaluation in MS, as CI is a common feature of MS, which direly impacts patients' quality of life, with 40–70% of patients affected at any time during the course of the disease.

**Rodica Balasa (Romania)**, the Head of the Neurology 1 Clinic at the Emergency Clinical County Hospital of Mures (Romania), ended the session with "Plasmatic exosomal microARN from brain cells in the evaluation of immunological treatment in multiple sclerosis patients". During her presentation, Multiple Sclerosis (MS) was discussed, its impact – as the leading cause of chronic neurological handicap in young adults – was showcased, and the difficulty in treatment due to its heterogeneity and the unknown aetiology as a probable result of the interplay of several different factors were touched upon. Prof. Balasa tackled the role of inflammation in demyelination and axonal loss, the processes of neurodegeneration and remyelination, and highlighted disease-modifying therapies, underlining that the best choice for the patient is based on the specific biological mechanism of CNS inflammation. As a result, the best-suggested response is precision medicine.

The last session of the day, Session 8, was introduced by Hari Shanker Sharma (Sweden) and Xianshuang Liu (USA). First, **Hari Shanker Sharma (Sweden)**, the Director of Research at the International Experimental Central Nervous System Injury & Repair (IECNSIR) from the University Hospital, Uppsala University (Sweden), presented "Methamphetamine exacerbates Alzheimer's disease pathology". Neuroprotective effects of nanowired Cerebrolysin with Neprilysin, discussing the particularities of the blood-brain barrier in the Alzheimer's disease and the effect of methamphetamine. As Alzheimer's disease (AD) represents a devastating neurological affection leading to lifetime disability, investigating the risk factors, which can accelerate the pathogenesis of AD, including substance abuse, is of utmost importance. Prof. Sharma also focused on the effects of methamphetamine intoxication on the spinal cord and the rapid morphological changes after acute intoxication, highlighting his research on the matter.

**Xianshuang Liu (USA)**, Associate scientist at the Department of Neurology from the Henry Ford Hospital, Detroit (USA), offered his presentation together with **Michel Chopp (USA)**, Vice Chairman for Research of the Department of Neurology and Scientific Director at the Henry Ford Neuroscience Institute, Detroit (USA), on "Exosomes enhance the coupling of neurogenesis and angiogenesis by transfer of microRNA", where the functions and uses of exosomes were explained, the process of neurogenesis and angiogenesis described, and the impact of stroke on their association was tackled.

**Ioana Mandruta (Romania)**, the Head of the Epilepsy Monitoring Unit at the University Emergency Hospital of Bucharest (Romania), presented "Semiology of epileptic seizures – the new glossary of terms". Prof. Mandruta discussed epilepsy, its subtypes, the classification of seizures, the need to address the terminology, and presented a case with epilepsy. She explained the definition of myoclonic jerks, offering examples, discussed seizures with focal onset and the appearance of seizure symptoms based on the affected part of the brain, and provided a glossary definition of elementary motor signs. Further on, Prof. Mandruta presented complex motor behaviors, subjective ictal symptoms, also called "aura", autonomic events, and the localization of seizure generators. Moreover, she exemplified another case of seizures with impaired consciousness and the exploration of the patient. Finally, she discussed temporal lobe epilepsy and posterior cortex epilepsy, ending with additional case studies on patients with epilepsy.

**Peter Jenner (UK)**, Emeritus Professor at the Institute Of Pharmaceutical Sciences from the Faculty of Life Sciences and Medicine at King's College in London (UK), presented "Can we develop non-dopaminergic approaches to the treatment of Parkinson's disease?" and began his lecture with a discussion on "breaking out of the dopaminergic box", highlighting the need for more complex approaches to PD and suggesting a multimodal pharmacological perspective as the best way in the future. Dopaminergic approaches dominate the field as they are highly effective for this disorder but can lead to complacency, considering there are still some needs in PD that cannot be treated with a dopaminergic approach. He suggested that although the dopaminergic treatment helps non-motor symptoms it is not the complete solution, and discussed safinamides as a treatment option. An essential point of his presentation was that there are non-dopaminergic components to both motor and non-motor symptoms of Parkinson's disease. **Cristian Falup-Pecurariu (Romania)**, the Head of the Department of Neurology at the County Clinic Hospital from Brasov (Romania), presented "Autonomic dysfunction in Parkinson's disease", discussing the spectrum of dysautonomia symptoms in PD and the prevalence of different symptoms (dependent on study methodology). Parkinson's Disease (PD) has a wide variety of non-motor symptoms and one of the frequently encountered ones – autonomic nervous system dysfunction – direly impacts the quality of patients' lives. In advanced stages, dysautonomia significantly impacts the activities of daily living. He then discussed the relationship between disease progression and survival rates of autonomic dysfunction and dysautonomia in patients with a high risk of PD, pinpointing on the autonomic dysfunction and disease severity. Methods of dysautonomia assessment and specific symptoms were additionally presented. Comprehensive approaches to treatment management were showcased.

**Alla Guekht (Russia)**, Director at the Moscow Research and Clinical Center for Neuropsychiatry in Moscow (Russia), presented "Post-stroke cognitive impairment and dementia – Treatment challenges", addressing epidemiology, the populations at risk, potential targets, prevention, treatment, and current strategies and perspectives. As the population is growing older, people 65 and over represent the fastest growing group, posing several issues, with post-stroke cognitive dysfunction representing an important factor of morbidity. With shifting demographics, increased post-stroke survival and life expectancy can also mean an increased number of patients with post-stroke cognitive impairment. Prof. Guekht discussed the importance of implementing results for clinical trials in different groups and addressing the challenges and unresolved issues in post-stroke cognitive decline. For this, the trajectory is of utmost importance. The complexity of the affection requires combined approaches and assessment at different time points, as well as consideration of the emotional and cognitive problems. She presented some risk factors that increase the magnitude and speed of post-stroke cognitive decline, highlighting early seizures after stroke and the post-stroke development of epilepsy. The relationship between stroke location and cognitive outcomes, the association of cognitive impairment with early seizures, and the multidomain lifestyle interventions to prevent cognitive decline after stroke were also subjects touched upon. Moreover, Prof. Guekht encouraged integrative approaches in diagnosing and treating neurological disorders and discussed the need for increased research and more clinical trials to address the best strategies for post-stroke cognitive impairment.

Next, Session 10, presided by Jozsef Szasz (Romania) and Cristian Falup Pecurariu (Romania) was introduced with a presentation on "Management of advanced Parkinson's disease with Levodopa intestinal gel" by **Jozsef Szasz (Romania)**, Associate Professor at the Department of Neurology at George Emil Palade University of Medicine, Pharmacy, Science and Technology of Targu Mures (Romania). He described the Romanian context for testing and initiating therapy by means of a multicenter study from 9 university teaching hospital centres on 113 patients. The presented research demonstrated the effects of therapy on patients with advanced Parkinson's disease. Prof. Szas detailed his research on the matter, highlighting, through video presentations of the patients over the years, the effects of the treatment.

**Dafin Mureșanu (Romania)**, the president of the European Federation of NeuroRehabilitation Societies (EFNR) and Chairman at the Department of Neurosciences from Iuliu Hatieganu University of Medicine and Pharmacy in Cluj-Napoca (Romania), then presented "Is neurorehabilitation useful in Parkinson's Disease?". Prof. Muresanu began with an introduction to brain functions and the shifting paradigm in neuroscience, offering a brief parallel between the physics and biology approaches in neuroscience. He further discussed the three circuitries of the nervous system, namely the limbic, the associative, and the sensorimotor, underlining the importance of said mechanisms working in harmony. Next, Prof. Muresanu masterfully described the "small-world" network organization of the brain, the endogenous modulation based on balancing anticorrelated processes, and the interrelation between the brain's levels of organization. Further on, he discussed basal ganglia and behavior regulation, the relationship between dopamine and executive dysfunction, and the relationship of rehabilitation with the biological reserve, highlighting the differences between the classic medical model and the rehabilitation model. Moreover, prof. Muresanu pinpointed the role of restoration *versus* compensation in neurorehabilitation, the specialists needed for developing a comprehensive rehabilitation team, and the functional rationale for neurorehabilitation in Parkinson's Disease. Prof. Muresanu also discussed guidelines for occupational therapy in PD, mentioned the importance of cueing in the rehabilitation process, and ended on a brief note on neurorehabilitation in different PD stages.

**Stanislav Groppa (Moldova)**, Head of Neurology Department at the State University of Medicine and Pharmacy Nicolae Testemitanu in Chisinau (Republic of Moldova) presented "Nonconvulsive Status Epilepticus – A Complex and Often an Under-Recognized Entity". Prof. Groppa discussed status epilepticus as a neurological emergency and a challenge of modern neurology, as the condition is difficult to delimit, and current clinical options remain unsatisfactory. This affection is often unrecognized, leading to delays in diagnosis and treatment, mostly due to failure to request an electroencephalogram (EEG). He showcased issues in diagnosis, the role of EEG on epidemiology, and pinpointed "borderline syndromes" where electrographic convulsive activity appears without obvious clinical seizures. The discussion then centered around the primary forms (nonconvulsive and convulsive), their causes, the trigger factors, clinical manifestations – including chameleon presentation and mimic disorders. Furthermore, he approached the management, complications, and treatment recommendations for this disorder, the need for tailored interventions, the importance of research with prospective studies on the effects of extended continuous EEG monitoring on outcomes and cost-effectiveness strategies for early identification and treatment.

The following session, Session 11, introduced by Adina Roceanu (Romania) and Corina Roman (Romania), began with a presentation by **Adina Roceanu (Romania)**, Primary Neurologist and Research Scientist at the Neurology Department from the University Emergency Hospital Bucharest (Romania), on migraine treatment. Beginning with the definition and symptoms of migraines, she offered insight into one of the most common affections for the larger population. Prof. Roceanu discussed the classifications of migraines with and without aura, highlighting specific criteria for each of the two presentations. She underlined related affections, including chronic migraines and medication-overuse headaches, while also showcasing the clinical phases of migraines and the role of specific agents in their treatment. Dr. Roceanu ended her presentation with a mention of some non-pharmacological approaches which are appropriate in situations where pharmacological treatment proves ineffective.

**Corina Roman (Romania)**, the Head of the Neurology Department at the County Hospital Sibiu (Romania), approached case presentations on secondary headaches. Prof. Roman discussed the international classification of headache disorders, the general principles, and the pharmacological agents that can cause headaches. She described red flags suggestive of secondary aetiology of headache in patient history and introduced clinical case presentations on headaches attributed to arteriovenous malformation, headaches caused by cerebral tumors, and patients with MS with associated secondary trigeminal neuralgia. Prof. Roman suggested that headache is prevalent and headache disorders are among the most common signs of nervous system disorders, making it vital to consider the patient history, neurological examinations, and the SNOOP mnemonic as the best predictors of pathology.

**Vitalie Vacaras (Romania)**, Lecturer of the Neurology and Pediatric Neurology Discipline at the Iuliu Hatieganu University of Medicine and Pharmacy in Cluj-Napoca (Romania) presented "When headache hides neurocysticercosis – Case presentations", an interesting clinical case with some unexpected characteristics. He began by discussing cysticercosis – what it is, what some of the risk factors and its most common manifestations are – and followed with the specific case, detailing the diagnostics and examination of the patient, the prognosis of the disease, initial treatment, evolution, and outcomes, discussing the implications and rationality for choosing the previously mentioned case.

The last session of the event, Session 12, presided by Catalin Jianu (Romania) and Vitalie Lisnic (Republic of Moldova), was introduced with the presentation of **Catalin Jianu (Romania)**, Professor of Neurology, Head of the First Department of Neurology, and Head of Advanced Research Centre for Cognitive Research in Neuropsychiatric Pathology at the Victor Babes University of Medicine and Pharmacy in Timisoara (Romania), "An integrated approach on the diagnosis of cerebral veins and dural sinuses thrombosis", in which Prof. Jianu began by offering the public information on the epidemiology of the disease. Then, he discussed cerebral veins anatomy, the anatomy of intracranial dural sinuses, the aetiology and risk factors, genetic and acquired thrombophilia, the most common risk factors, and the impact of COVID-19. Next, he covered the pathophysiological mechanisms leading to the clinical presentation of cerebral venous thrombosis (CVT), and also discussed clinical diagnosis, the importance of laboratory data and tests, and imaging methods. Further on, Prof. Jianu presented the experience of the hospitals, an analysis of 70 patients admitted between 1998 and 2021 and the results, as well as a review of an integrated approach to the diagnosis of cerebral veins and dural sinuses thrombosis. He then showcased another case review on cavernous sinus thrombosis in a woman with inherent thrombophilia and one on acute isolated lateral sinus thrombosis. One important takeaway from his presentation was that a comprehensive clinical spectrum with an often-misleading presentation might cause diagnosis delays.

**Mihail Gavriliuc (Republic of Moldova)**, Professor of Neurology and Chairman of the Neurology Department from the State University of Medicine and Pharmacy Nicolae Testemitanu in Chisinau (Republic of Moldova), presented on Neuroborreliosis, an antropozoonosis also known as Lyme disease. Prof. Gavriliuc discussed the aetiology of different species of Borrelia, the biological basis and different presentations, Lyme disease vectors, and the deer tick's life cycle. He approached epidemiology, pathogenesis and pathophysiology, and clinical manifestations for early and late neuroborreliosis. He highlighted the Bannwarth triad symptoms and outlined differential diagnosis of Lyme neuroborreliosis, diagnostic workup, the management of the disease in the early and late stages and the importance of individualized therapies.

**Vitalie Lisnic (Republic of Moldova)**, Professor of Neurology at the Nicolae Testemitanu State University of Medicine and Pharmacy in Chisinau (Republic of Moldova), ended the congress with his presentation "Wilson disease – a constellation of phenotypes". First, he discussed the history of the disease, its aetiology, prevalence, and other epidemiological aspects and highlighted the clinical picture and the existence of a delay in diagnosis. Further on, Prof. Lisnic described hepatic, psychiatric, ophthalmic, and neurological manifestations, followed by methods of diagnosis. Lastly, he presented clinical cases and treatment with different cure options for various presentations and discussed new treatment avenues. One fascinating insight of his proceedings is an algorithm for treating Wilson's disease. He ended by stating the importance of investigating Wilson Disease (WD) in any patient with neuropsychiatric manifestations of unknown cases. The disease can be fatal without therapy, yet treatment has an excellent prognosis.

The conclusions of the event were drawn by Dafin Mureșanu (Romania), Natan Bornstein (Israel), Volker Homberg (Germany), and Wolfgang Grisold (Austria), with Prof. Muresanu sharing his hopes for an in-person next edition of the Summer School of Neurology. Prof. Muresanu touched upon the possibility of future hands-on programs and extended his gratitude to everyone who contributed to the development of the program.

